# Real-world patient-reported outcomes and concordance between patient and physician reporting of side effects across lines of therapy in multiple myeloma within the USA

**DOI:** 10.1007/s00520-023-07836-x

**Published:** 2023-06-02

**Authors:** Amanda Ribbands, Natalie Boytsov, Abigail Bailey, Boris Gorsh, Emily Luke, Annabel Lambert

**Affiliations:** 1 Oncology Franchise, Adelphi Real World, Bollington, UK; 2 GSK, Collegeville, PA USA

**Keywords:** Health-related quality of life, Patient-physician communication, Patient-physician concordance, Patient-reported outcomes, Multiple myeloma, Side effects

## Abstract

**Purpose:**

We aimed to explore patient-reported outcomes (PROs) and patient and physician concordance of side effects perception across lines of therapy (LOT) in multiple myeloma (MM) within the United States of America (USA).

**Methods:**

Data were drawn from the Adelphi Real World MM III Disease Specific Programme™, a point-in-time survey of hemato-oncologists/hematologists and their patients with MM conducted in the USA between August 2020 and July 2021. Physicians reported patient characteristics and side effects. Patients reported side-effect bother and health-related quality of life (HRQoL) using validated PRO tools (European Organisation for the Research and Treatment of Cancer Quality of Life Core Questionnaire/-MM Module [EORTC QLQ-C30/-MY20], EQ-5D-3L and Functional Assessment of Cancer Therapy—General Population physical item 5). Descriptive, linear regression and concordance analyses were performed.

**Results:**

Records from 63 physicians and 132 patients with MM were analyzed. EORTC QLQ-C30/-MY20 and EQ-5D-3L scores were consistent across LOTs. Scores tended to be worse with higher side-effect bother; patients “very much” bothered by side effects had lower median (interquartile range) global health status scores (33.3 [25.0–50.0]) than those “not at all” bothered (79.2 [66.7–83.3]). Patient and physician concordance on side-effect reporting was poor to fair. Patients frequently reported fatigue and nausea as bothersome side effects.

**Conclusion:**

HRQoL of patients with MM was worse with greater side-effect bother. Discordant patient and physician reporting of side effects indicated a need for improved communication during management of MM.

**Supplementary information:**

The online version contains supplementary material available at 10.1007/s00520-023-07836-x.

## Introduction

Multiple myeloma (MM) is the second most common hematological malignancy in the United States of America (USA) [1]. MM is associated with poor health-related quality of life (HRQoL), particularly in older patients, and affects multiple physiological systems, leading to symptoms such as bone lesions, pain, and repeated infections [1–3]. MM remains incurable, but the development of novel therapies has enabled effective management and led to improved survival [2, 4].

Most patients require long-term treatment associated with side effects that may negatively affect their HRQoL [2]. Physicians selecting treatments to maximize survival for individual patients should also consider a range of patient characteristics, such as health status and age, prior treatments, cumulative toxicities, anticipated adverse events, and MM-related symptoms [5]. Treatment profiles can then be weighed against each other in terms of multiple outcomes, leading to more personalized treatment selection. However, individual patient profiles may be too complex to allow full consideration of all relevant factors, especially in patients who are already refractory to multiple treatment classes, adding to the complexity of treatment choice [6].

Due to the complexity of possible treatment pathways for patients with MM, it is essential to incorporate patient perspectives on their own health status and preferred treatment outcomes in shared clinical decision-making [7]. For example, as treatment-related toxicities may become more burdensome than MM symptoms [8], it is important to balance the potential efficacy of a treatment against its potential side effects to achieve patients’ desired clinical and HRQoL outcomes. Therefore, physicians need to understand their patients’ priorities to determine the appropriate profile of benefits and risks of treatment. Together with physicians’ expertise, the inclusion of patient preferences in treatment selection is fundamental for improving treatment adherence and outcomes [7, 8].

Real-world evidence (RWE) has demonstrated the complexity of MM treatment choice, particularly as patients progress through multiple lines of therapy (LOT) [9, 10]. With each successive LOT, treatment options for patients with MM have become increasingly complex due to the number of available regimens for patients with relapsed/refractory MM (RRMM) [4]. Studies have shown that in real-world practice, physicians tend to have a greater focus on survival outcomes when making treatment choices, with less consideration of other factors such as side effects and the need for patient visits [11]. Although patients also prioritize survival, they place higher importance on additional factors known to influence HRQoL, such as minimizing treatment side effects and offsetting financial burdens [12]. However, it is currently unclear to what extent patients’ HRQoL changes over the course of their treatment, and the level of concordance between patients’ and physicians’ reporting of treatment side effects.

The aim of the present study was to describe patient-reported outcomes (PROs) among patients treated for MM in the USA and provide insight into the extent of concordance between patient- and physician-reported side effects across LOTs.

## Methods

### Study design

This study drew data from the Adelphi Real World (ARW) MM III Disease Specific Programme (DSP)™, a point-in-time, multi-sponsor survey of physicians and their consulting patients with MM in the USA between August 2020 and July 2021. The DSP methodology has been previously described and validated [13–15].

Data were collected from four main DSP sources: (I) patient record forms, detailed records completed by the physician for the next 8 consecutively seen patients who met the inclusion criteria; (II) a short survey capturing physician demographics; (III) a physician workload survey completed by the physician for each day over a 5-day period to record the number of patients they consulted with; and (IV) voluntary patient self-completion questionnaires, filled by patients independently of their physician immediately after consultation. Completion of the patient questionnaires was voluntary, so not all PRO measures were completed by all patients. The matched patient self-completion questionnaires with corresponding patient record forms comprised the final database for analysis.

### Study population

Eligible hemato-oncologists and hematologists recruited into the ARW MM III DSP survey were physicians across the USA who were actively involved in prescribing decisions for patients with RRMM. Physicians were eligible for inclusion if they saw a minimum of 6 unique patients with RRMM per month, although a degree of flexibility was applied to ensure that physicians with lower caseloads were considered.

The patient population was a convenience sample comprising the next 8 eligible patients with MM seen by each enrolled physician across different lines of active drug therapy. These 8 patients comprised a quota of 2 patients each receiving their first LOT (1L), second LOT (2L), third LOT (3L), and fourth or later LOT (4L +). Eligible patients were ≥ 18 years of age, had a confirmed diagnosis of MM, and had been receiving an active drug treatment at time of data collection. Patients who participated in a clinical trial or who were no longer receiving an active systemic drug treatment for their MM (i.e. receiving palliative care only) were excluded from the study.

### Outcomes

Physicians reported patients’ characteristics including demographics (age at data collection, sex and ethnicity), clinical characteristics (cytogenetic risk, Eastern Cooperative Oncology Group [ECOG] score and International Staging System [ISS] stage at diagnosis), treatment history (current and prior LOTs at data collection, including refractory status), outcomes associated with each treatment used, and side effects. Patients were considered refractory to treatment when their disease became non-responsive (defined as failure of treatment to achieve at least a minimal response) or progressive either on therapy or within 60 days of last treatment. Patients reported their perceptions of MM treatment and side effects they had experienced, including information on HRQoL, global health status, functional scores, body image, MM symptoms, level of side-effect bother, perspective on the future, and financial impact of MM.

To assess key aspects of patients’ HRQoL that may have been impacted by MM, patients completed the European Organisation for the Research and Treatment of Cancer Quality of Life Core Questionnaire (EORTC QLQ-C30) and the EORTC MM Module (EORTC QLQ-MY20). To evaluate core dimensions of overall HRQoL and health state, patients completed the descriptive self-assessed EQ-5D-3L. Patients also reported the level of bother associated with side effects they experienced as a result of MM treatment using item 5 on the physical well-being scale of the Functional Assessment of Cancer Therapy—General Population questionnaire (FACT-GP5; 5-point Likert scale). Differences in HRQoL scores between patients at different LOTs were assessed with respect to published minimal important differences (MID) for each PRO measure [16–18]. Changes above MID were considered clinically important. Further details of each PRO are provided within the Supplementary Materials.

### Data analysis

Descriptive statistics (median, interquartile range [IQR] and frequency) were calculated for demographic and clinical characteristics. All PROs were scored according to the published guidelines [16–19] and analyzed descriptively. Categorical variables were presented as frequency and percentage where appropriate. Ordinal variables were reported as frequency and percentage and/or median (IQR), as appropriate for the individual variables. Continuous variables were reported as median (IQR) and range. Variables were analyzed as observed, with no imputation of missing data or aggregation across questions.

EORTC QLQ-C30 and EORTC QLQ-MY20 data were analyzed using linear regression with covariates that were selected based on their previously identified influence on HRQoL [20, 21]: age (< 65 vs ≥ 65), sex (male vs female), LOT (1L vs 2L; 1L vs 3L; 1L vs 4L), side-effect bother (0 [Not at all] vs 1 [A little bit]; 0 [Not at all] vs 2 [Somewhat]; 0 [Not at all] vs 3 [Quite a bit] or 4 [Very much]) and Eastern Cooperative Oncology Group scores at data collection (0 vs 1; 0 vs 2 +) [20, 21]. Coefficients were estimated for each covariate, which indicate numeric change in the outcome per unit increase in the covariate, and reported with the associated P-value and 95% confidence interval.

Concordance analyses using Cohen’s kappa coefficient (κ) were conducted to determine the agreeability between patient- and physician-reported side effects of MM at time of data collection, and to evaluate the extent of agreement between physician-reported side effect severity and patient-reported level of side-effect bother. Interpretation of κ was based on the following criteria: κ < 0, poor agreement; κ = 0.01–0.2, slight agreement; κ = 0.21–0.4, fair agreement; κ = 0.41–0.6, moderate agreement; κ = 0.61–0.8, substantial agreement; κ = 0.81–1.0, almost perfect agreement [22]. Side effects were categorized into: gastrointestinal (GI; nausea, diarrhea, constipation and vomiting); hematological/circulatory (anemia, thrombocytopenia, neutropenia, leucopenia and thrombosis); neurological/psychological (neuropathy, depression and mood changes); dermatological (rash and dry skin); and other (fatigue, change in appetite, weight change, blurred vision, dry eye, headaches, hair loss, infections, general aches and pains, fever/flu-like symptoms and mouth sores).

## Results

### Demographics and clinical characteristics

Participating physicians (n = 63) completed patient record forms for 377 patients. Physicians were almost equally split between academic (44%) and community (56%) practice. Participating physicians managed a median of 52 (range: 20–400) patients with MM and 70% had current or prior involvement in clinical trials in MM.

A total of 132 patients (35%) had matched physician-completed patient record forms and corresponding patient self-completion questionnaires. Of these, 40 patients (30%) were at 1L, 32 (24%) were at 2L, 31 (24%) were at 3L, and 29 (22%) were at 4L at time of data collection.

The matched patient study population (*n* = 132) were predominantly male (65%), white (70%), retired (67%), and had Medicare health insurance (60%); median (IQR) age was 70.0 (63.2–73.0) years (Table S1 in the Online Resource). The median (IQR) time since diagnosis for all patients with a known date of MM diagnosis (*n* = 117) was 24.3 (5.8–38.4) months. At time of MM diagnosis, 49% (65/132) of patients had International Staging System stage II disease. Among 67 patients who were tested, 37% had high-risk cytogenetic features, defined as a positive test for del(17p), t(4;14), t(14;16), del(17/17p), or gain(1q) genetic abnormalities [23].

At the time of data collection, the majority of patients had received triplet regimens in current and prior LOTs (1L: 79%, 2L: 80%, 3L: 62%, 4L: 33%). Regimens including proteasome inhibitors, immunomodulatory agents and CD38-targeted treatments were used across all LOTs, with the greatest frequency in earlier relapsed/refractory settings. Of the 23 (17%) patients who were triple-class exposed, 5 (22%) were refractory, 11 (48%) were not refractory and 7 (30%) had unknown refractory status.

### Patients’ health status and quality of life at time of data collection

**Fig. 1:**
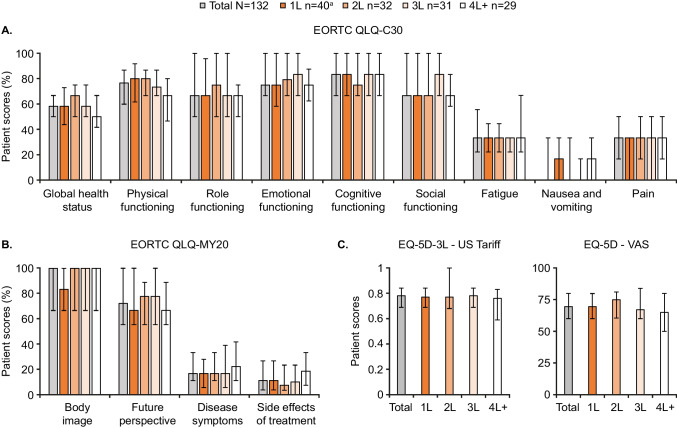
Patient scores for (**A**) EORTC QLQ-C30, (**B**) EORTC QLQ-MY20, and (**C**) EQ-5D-3L, stratified by LOT. Data are presented as median ± IQR. EORTC QLQ-C30/-MY20 scores range from 0.0–100.0; high scores for global health status represent high quality of life, high scores for functional scales represent high/healthy level of functioning, and high scores for symptoms scales represent high level of symptomatology. The EQ-5D-3L utility index ranges from 0.0 (dead) to 1.0 (full health), with values less than 0.0 being possible for states worse than dead; a clinically meaningful score change is regarded as ≥ 0.08 points. The EQ-5D VAS ranges from 100.0 to 0.0; higher values indicate better perceived HRQoL, and a clinically meaningful score change is regarded as ≥ 7 points or more. ^a^One patient in the 1L group did not complete the EQ-5D-3L questionnaire. 1L, first LOT; 2L, second LOT; 3L, third LOT; 4L, fourth LOT; EORTC QLQ-C30, European Organisation for the Research and Treatment of Cancer Quality of Life Core Questionnaire; EORTC QLQ-C30/-MY20, European Organisation for the Research and Treatment of Cancer Quality of Life Core Questionnaire/-MM Module; HRQoL, health-related quality of life; IQR, interquartile range; LOT, line(s) of therapy; US, American; VAS, Visual Analogue Scale

PRO scores for the overall population (*n* = 132) and by each LOT are shown in Fig. 1 and Table 1. Median EORTC QLQ-C30 global health status score ranged from 66.7 at 2L to 50.0 at 4L. All comparisons between 4L and earlier LOTs exceeded the MID, although it should be noted that these numerical comparisons were not adjusted for patient baseline characteristics. For all other EORTC QLQ-C30 domains except fatigue, pain, and diarrhea, clinically meaningful differences were observed between at least 2 LOTs. Patients receiving 1L had clinically meaningfully lower EORTC QLQ-MY20 scores for body image than those receiving later LOTs. Scores for 2L, 3L, and 4L were similar. Future perspective scores were lower in 1L and 4L than in 2L and 3L, and clinically meaningful difference in side effects of treatment was observed between patients at 2L and 4L. EQ-5D-3L—US Tariff scores were similar, with no clinically meaningful differences observed across LOTs. For EQ-5D visual analogue scale (VAS), clinically meaningful differences were observed between patients in 2L and 3L, and those in 2L and 4L.

**Table 1:** PRO scores stratified by LOT

	LOT			
	1L *n* = 40	2L *n* = 32	3L *n* = 31	4L *n* = 29
EORTC QLQ-C30 scores per item, median (IQR)				
Global health status	58.3 (43.8–72.9)	66.7 (50.0–75.0)	58.3 (50.0–75.0)	50.0 (41.7–66.7)
Physical functioning	80.0 (61.7–91.7)	80.0 (66.7–86.7)	73.3 (66.7–86.7)	66.7 (46.7–80.0)
Role functioning	66.7 (66.7–95.8)	75.0 (50.0–100.0)	66.7 (50.0–100.0)	66.7 (50.0–75.0)
Emotional functioning	75.0 (58.3–100.0)	79.2 (66.7–100.0)	83.3 (66.7–100.0)	75.0 (62.5–87.5)
Cognitive functioning	83.3 (66.7–100.0)	75.0 (66.7–100.0)	83.3 (66.7–100.0)	83.3 (66.7–100.0)
Social functioning	66.7 (66.7–100.0)	66.7 (66.7–100.0)	83.3 (66.7–100.0)	66.7 (58.3–83.3)
Fatigue	33.3 (22.2–44.4)	33.3 (22.2–44.4)	33.3 (22.2–33.3)	33.3 (22.2–66.7)
Nausea and vomiting	16.7 (0.0–33.3)	0.0 (0.0–33.3)	0.0 (0.0–16.7)	16.7 (0.0–33.3)
Pain	33.3 (0.0–33.3)	33.3 (16.7–50.0)	33.3 (0.0–50.0)	33.3 (16.7–50.0)
Dyspnea	33.3 (0.0–33.3)	0.0 (0.0–33.3)	0.0 (0.0–33.3)	0.0 (0.0–33.3)^a^
Insomnia	16.7 (0.0–33.3)	16.7 (0.0–33.3)	33.3 (0.0–33.3)	33.3 (16.7–50.0)
Appetite loss	33.3 (0.0–33.3)	33.3 (0.0–58.3)	0.0 (0.0–33.3)	33.3 (0.0–33.3)
Constipation	0.0 (0.0–33.3)	0.0 (0.0–0.0)	0.0 (0.0–33.3)	33.3 (0.0–33.3)
Diarrhea	0.0 (0.0–33.3)	0.0 (0.0–33.3)	0.0 (0.0–33.3)	0.0 (0.0–33.3)
Financial difficulties	33.3 (0.0–33.3)	33.3 (0.0–33.3)	33.3 (0.0–33.3)	33.3 (0.0–33.3)
EORTC QLQ-MY20 scores per item, median (IQR)				
Body image	83.3 (66.7–100.0)	100.0 (66.7–100.0)	100.0 (66.7–100.0)	100.0 (66.7–100.0)
Future perspective	66.7 (55.6–100.0)	77.8 (55.6–88.9)	77.8 (55.6–100.0)	66.7 (55.6–88.9)
Disease symptoms	16.7 (5.6–27.8)	16.7 (11.1–33.3)	16.7 (5.6–38.9)	22.2 (11.1–41.7)
Side effects of treatment	11.1 (3.7–26.7)	7.4 (3.3–23.3)	10.0 (0.7–0–23.3)	18.5 (7.4–33.3)
EQ-5D-3L utility (US Tariff) and health state (VAS) scores, median (IQR)				
EQ-5D-3L—US Tariff	0.77 (0.7–0.8)	0.77 (0.7–1.00)^b^	0.78 (0.69–0.84)	0.76 (0.59–0.83)
EQ-5D VAS	69.5 (60.0–79.8)	75.0 (60.5–81.0)	67.0 (60.0–84.0)	65.0 (50.0–80.0)

EORTC QLQ-C30/-MY20 scores range from 0.0–100.0; high scores for global health status represent high quality of life, high scores for functional scales represent high/healthy level of functioning, and high scores for symptoms scales represents high level of symptomatology

The EQ-5D-3L utility index ranges from 0.0 (dead) to 1.0 (full health), with values less than 0.0 being possible for states worse than dead; a clinically meaningful score change is regarded as one of 0.08 points or more

The EQ-5D VAS ranges from 100.0 to 0.0; higher values indicate better perceived health status, and a clinically meaningful score change is regarded as one of 7 points or more

^a^data from *n* = 28; ^b^data from *n* = 39

1L, first LOT; 2L, second LOT; 3L, third LOT; 4L, fourth LOT; EORTC QLQ-C30, European Organisation for the Research and Treatment of Cancer Quality of Life Core Questionnaire; EORTC QLQ-C30/-MY20, European Organisation for the Research and Treatment of Cancer Quality of Life Core Questionnaire/-MM Module; IQR, interquartile range; LOT, line(s) of therapy; PRO, patient-reported outcome; US, American; VAS, Visual Analogue Scale

**Fig. 2:**
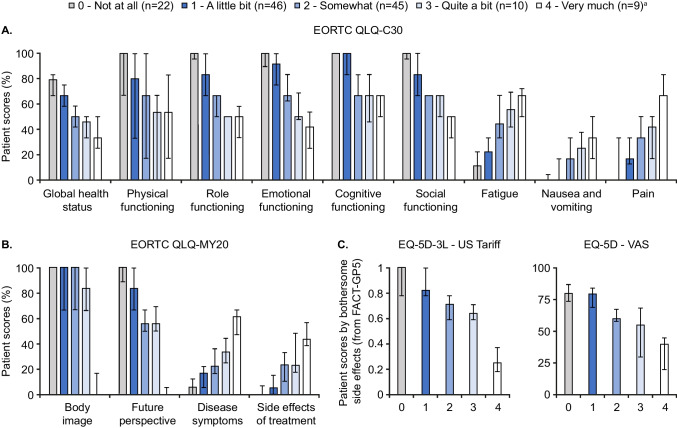
Patient scores for (**A**) EORTC QLQ-C30, (**B**) EORTC QLQ-MY20, and (**C**) EQ-5D-3L, stratified by FACT-GP5 bothersome side effects. Data are presented as median ± IQR. EORTC QLQ-C30/-MY20 scores range from 0.0–100.0; high scores for global health status represent high quality of life, high scores for functional scales represent high/healthy level of functioning, and high scores for symptoms scales represents high level of symptomatology. The EQ-5D-3L utility index ranges from 0.0 (dead) to 1.0 (full health), with values less than 0.0 being possible for states worse than dead; a clinically meaningful score change is regarded as ≥ 0.08 points. The EQ-5D VAS ranges from 100.0 to 0.0; higher values indicate better perceived health status, and a clinically meaningful score change is regarded as one of 7 points or more. ^a^One patient in the “4—Very much” group did not complete the EQ-5D-3L—US Tariff questionnaire (*n* = 8). EORTC QLQ-C30, European Organisation for the Research and Treatment of Cancer Quality of Life Core Questionnaire; EORTC QLQ-C30/-MY20, European Organisation for the Research and Treatment of Cancer Quality of Life Core Questionnaire/-MM Module; FACT-GP5, Functional Assessment of Cancer Therapy—General Population, item 5; IQR, interquartile range; US, American; VAS, Visual Analogue Scale

Figure 2 and Table 2 show PRO scores stratified by level of side-effect bother assessed using FACT-GP5. When stratifying EORTC QLQ-C30 scores by side-effect bother, global health status and functioning scores tended to be worse with higher levels of bother. Higher levels of bother were generally observed with higher severity of fatigue, nausea and vomiting, and pain. For global health status, clinically meaningful differences were observed between all levels of side-effect bother, except between patients who reported being “somewhat” bothered by side effects and those bothered “quite a bit”. In most cases, clinically meaningful differences were also reported between incrementally higher levels of side-effect bother for all other EORTC QLQ-C30 domains. EORTC QLQ-MY20 scores for disease symptoms and treatment side effects tended to be higher with higher levels of bother, whereas the opposite was seen for body image and future perspectives scores, with clinically meaningful differences also observed between most levels of side-effect bother. EQ-5D-3L—US Tariff and EQ-5D VAS scores were lower with higher levels of bother; in both cases, clinically meaningful differences were observed between all levels of side-effect bother except between patients who were “somewhat” and “quite a bit” bothered by side effects.

**Table 2:** PRO scores in total and stratified by bothersome side effects (from FACT-GP5)

	Bothersome side effects					
	Total *N* = 132	0—Not at all *n* = 22	1—A little bit *n* = 46	2—Somewhat *n* = 45	3—Quite a bit *n* = 10	4—Very much *n* = 9
EORTC QLQ-C30 scores per item, median (IQR)						
Global health status	58.3 (50.0–66.7)	79.2 (66.7–83.3)	66.7 (58.3–75.0)	50.0 (41.7–58.3)	45.8 (33.3–50.0)	33.3 (25.0–50.0)
Physical functioning	76.7 (60.0–86.7)	100.0 (86.7–100.0)	80.0 (73.3–93.3)	66.7 (53.3–80.0)	53.3 (43.3–61.7)	53.3 (40.0–66.7)
Role functioning	66.7 (50.0–100.0)	100.0 (95.8–100)	83.3 (66.7–100)	66.7 (50.0–66.7)	50.0 (50.0–50.0)	50.0 (33.3–58.3)
Emotional functioning	75.0 (66.7–100.0)	100.0 (89.6–100)	91.7 (75.0–100.0)	66.7 (62.5–83.3)	50.0 (47.9–68.8)	41.7 (25.0–54.2)
Cognitive functioning	83.3 (66.7–100.0)	100.0 (100.0–100.0)	100.0 (83.3–100.0)	66.7 (66.7–83.3)	66.7 (45.8–83.3)	66.7 (50.0–66.7)
Social functioning	66.7 (66.7–100.0)	100.0 (95.8–100)	83.3 (66.7–100)	66.7 (66.7–66.7)	66.7 (50.0–66.7)	50.0 (33.3–50.0)
Fatigue	33.3 (22.2–55.6)	11.1 (0.0–22.2)	22.2 (22.2–33.3)	44.4 (33.3–66.7)	55.6 (41.7–69.4)	66.7 (50.0–72.2)
Nausea and vomiting	0.0 (0.0–33.3)	0.0 (0.0–4.2)	0.0 (0.0–16.7)	16.7 (0.0–33.3)	25.0 (0.0–37.5)	33.3 (16.7–50.0)
Pain	33.3 (16.7–50.0)	0.0 (0.0–33.3)	16.7 (12.5–33.3)	33.3 (33.3–50.0)	41.7 (16.7–50.0)	66.7 (66.7–83.3)
Dyspnea	0.0 (0.0–33.3)	0.0 (0.0–0.0)	0.0 (0.0–33.3)	33.3 (0.0–66.7)	33.3 (0.0–33.3)	33.3 (8.3–66.7)
Insomnia	33.3 (0.0–33.3)	0.0 (0.0–8.3)	0.0 (0.0–33.3)	33.3 (16.7–33.3)	33.3 (33.3–66.7)	66.7 (33.3–100)
Appetite loss	33.3 (0.0–33.3)	0.0 (0.0–0.0)	0.0 (0.0–33.3)	33.3 (16.7–33.3)	50.0 (25.0–66.7)	66.7 (50.0–66.7)
Constipation	0.0 (0.0–33.3)	0.0 (0.0–0.0)	0.0 (0.0–0.0)	33.3 (0.0–33.3)	16.7 (0.0–33.3)	33.3 (16.7–66.7)
Diarrhea	0.0 (0.0–33.3)	0.0 (0.0–0.0)	0.0 (0.0–33.3)	0.0 (0.0–33.3)	33.3 (25.0–66.7)	33.3 (33.3–50.0)
Financial difficulties	33.3 (0.0–33.3)	0.0 (0.0–0.0)	0.0 (0.0–33.3)	33.3 (0.0–33.3)	33.3 (33.3–41.7)	100.0 (83.3–100.0)
EORTC QLQ-MY20 scores per item, median (IQR)						
Body image	100.0 (66.7–100.0)	100.0 (100.0–100.0)	100.0 (66.7–100.0)	100.0 (66.7–100.0)	83.3 (66.7–100.0)	0.0 (0.0–16.7)
Future perspective	72.2 (55.6–100.0)	100.0 (88.9–100.0)	83.3 (66.7–100.0)	55.6 (50.0–66.7)	55.6 (50.0–69.4)	0.0 (0.0–5.6)
Disease symptoms	16.7 (11.1–33.3)	5.6 (0.0–12.5)	16.7 (5.6–22.2)	22.2 (16.7–36.1)	33.3 (25.0–44.4)	61.1 (47.2–66.7)
Side effects of treatment	11.1 (3.7–26.7)	0.0 (0.0–6.9)	5.2 (0.0–15.3)	23.3 (10.6–33.3)	22.8 (17.6–48.3)	43.3 (38.5–56.7)
EQ-5D-3L utility (US Tariff) and health state (VAS) scores, median (IQR)						
EQ-5D-3L—US Tariff	0.78 (0.69–0.84)	1.00 (0.78–1.0)	0.82 (0.78–1.0)	0.71 (0.59–0.78)	0.64 (0.59–0.71)	0.25 (0.18–0.37)
EQ-5D VAS	69.5 (60.0–80.0)	80.0 (73.8–87.0)	79.5 (69.0–84.2)	60.0 (58.0–67.5)	55.0 (29.8–68.5)	40.0 (20.0–45.0)

EORTC QLQ-C30/-MY20 scores range from 0.0–100.0; high scores for global health status represent high quality of life, high scores for functional scales represent high/healthy level of functioning, and high scores for symptoms scales represents high level of symptomatology. The EQ-5D utility index ranges from 0.0 (dead) to 1.0 (full health), with values less than 0 being possible for states worse than dead; a clinically meaningful score change is regarded as one of 0.08 points or more. The EQ-5D VAS ranges from 100.0 to 0.0; higher values indicate better perceived health status, and a clinically meaningful score change is regarded as one of 7 points or more

1L, first LOT; 2L, second LOT; 3L, third LOT; 4L, fourth LOT; EORTC QLQ-C30, European Organisation for the Research and Treatment of Cancer Quality of Life Core Questionnaire; EORTC QLQ-C30/-MY20, European Organisation for the Research and Treatment of Cancer Quality of Life Core Questionnaire/-MM Module; FACT-GP5, Functional Assessment of Cancer Therapy—General Population item 5; IQR, interquartile range; LOT, line(s) of therapy; PRO, patient-reported outcome; US, American; VAS, Visual Analogue Scale

**Fig. 3:**
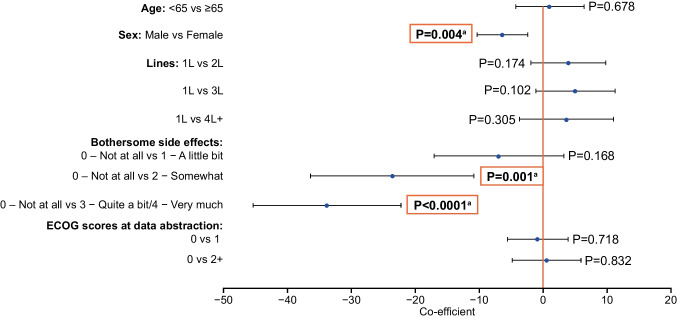
Linear regression analysis of EORTC QLQ-C30: Global health status. Data are presented as the estimate coefficient ± 95% CI for each covariate. ^a^Indicates statistically significant P-value. Significant values < 0.0 indicate a higher likelihood of having a lower global health status score. 1L, first LOT; 2L, second LOT; 3L, third LOT; 4L, fourth LOT; CI, confidence interval; ECOG, Eastern Cooperative Oncology Group; EORTC QLQ-C30, European Organisation for the Research and Treatment of Cancer Quality of Life Core Questionnaire

In a linear regression analysis of EORTC QLQ-C30 global health status scores where all other categories were fixed, female patients were significantly more likely to have a lower health status score than male patients (average difference of 6.4; *P* = 0.004; Fig. 3). When compared with patients who reported being “not at all” bothered by treatment side effects, patients who reported being “somewhat” bothered were significantly more likely to have a lower EORTC QLQ-C30 score (average difference of 23.6; *P* = 0.001), as were patients who reported being “quite a bit/very much” bothered by side effects (average difference of 33.8; *P* < 0.0001). Similar findings for side-effect bother were observed in linear regression analyses of the EORTC QLQ-C30 functional scale scores (shown in Fig. S1 in the Online Resource).

In a linear regression analysis of EORTC QLQ-MY20 body image scale where all other categories were fixed, female patients were more likely to have a lower score than male patients (average difference of 11.5; *P* = 0.026); 3L and 4L + patients were both more likely to have a higher score than 1L patients (average difference of 13.8; *P* = 0.21 and *P* = 0.004, respectively). When compared with patients who reported being “not at all” bothered by treatment side effects, patients who reported being “somewhat bothered” were more likely to have a lower EORTC QLQ-MY20 body image score (average difference of 19.2; *P* = 0.02), as were patients who reported being “quite a bit/very much bothered” by side effects (average difference of 52.9; *P* = 0.022). Similar findings for side-effect bother were observed in linear regression analyses of the other EORTC QLQ-MY20 elements (Fig. S2 in the Online Resource).

### Patient- and physician-reported side effects associated with treatment received at time of data collection

**Fig. 4:**
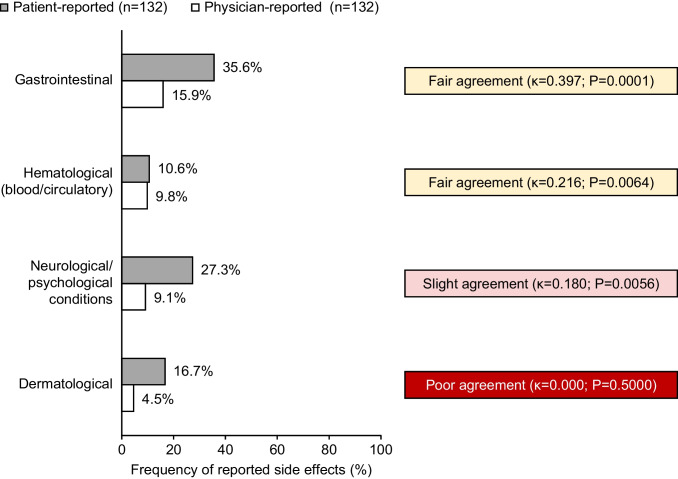
Total patient- and physician-reported side effects at data collection. Data show number of patient self-completion questionnaires and physician-reported patient record forms reporting each class of side effects. Extent of agreement was determined using Cohen’s kappa coefficient (κ)

Concordance between patients and physicians on the frequency of side effects reported across all LOTs ranged from poor to fair (Figs. 4 and S3 in the Online Resource). Concordance analysis indicated fair agreement between patients and physicians for GI (κ = 0.397; *P* = 0.0001) and hematological (κ = 0.216; *P* = 0.0064) side effects; neurological/psychological side effects showed slight agreement (κ = 0.180; *P* = 0.0056) while dermatological side effects had poor agreement (κ = 0.000; *P* = 0.5000). Patients typically reported a higher incidence of GI, neurological/psychological, and dermatological side effects compared with physicians.

Overall, GI side effects were the most common category of side effect reported by both patients (47/132 patients who completed the relevant questionnaire, 36%) and physicians (for 40/73 patients whose physicians reported any side effects, 55%). Across all LOTs, GI side effects appeared to be reported most frequently for patients at 4L by both patients (1L: 38%, 2L: 31%, 3L: 26%, 4L: 48%) and physicians (1L: 44%, 2L: 53%, 3L: 43%, 4L: 79%), although patient numbers in each LOT were small (*n* = 29–40) (Table S2 in the Online Resource). Regarding specific side effects, fatigue was the most commonly reported by both patients (57/132 patients, 43%) and physicians (26/73, 36%), followed by nausea (patients: 33/132, 25%; physicians: 24/73, 33%). Compared with other LOTs, nausea appeared to be reported most frequently at 4L by patients (1L: 28%, 2L: 19%, 3L: 19%, 4L: 34%) and physicians (1L: 32%, 2L: 33%, 3L: 14%, 4L: 47%) (Table S2 in the Online Resource).

Patients frequently reported fatigue/tiredness (57/132, 43%), nausea (33/132, 25%) and general aches and pains (22/132, 17%) as bothersome side effects; these side effects were generally rated on FACT-GP5 as “a little bit/somewhat” bothersome (fatigue: 44/57, 77%; nausea: 21/33, 64%; aches and pains: 11/22, 50%). In those patients who reported being “quite a bit/very much” bothered by their side effects, diarrhea (5/7, 71%) and flushing (5/5, 100%) were the most bothersome (Table S3 in the Online Resource).

## Discussion

This point-in-time study used RWE to examine HRQoL outcomes in patients with MM, the influence of treatment side effects on HRQoL, and concordance between patients and physicians when reporting side effects. We found that patients’ global health status, functioning, and MM symptom scores were generally similar across groups of patients receiving different LOTs; however, HRQoL appeared worse in those with higher levels of side-effect bother. Concordance analysis showed that agreement between patients and physicians on the frequency of GI and hematological side effects reported across all LOTs was fair, although patients typically reported a higher incidence of GI, dermatological, and neurological/psychological side effects than physicians. Across all LOTs, GI was the most common side-effect category, with fatigue followed by nausea being the most common specific side effects reported by both physicians and patients. Patients frequently reported fatigue and nausea as bothersome side effects.

With the continued advancement of MM treatment therapies, a growing population of patients with RRMM have accumulated physical and psychosocial burdens, or “late effects”, from both MM and successive LOTs [3]. Such late effects can detrimentally affect patient experience in cancer treatment, especially in patients with RRMM given their extended post-diagnosis survival and prolonged disease control in the absence of a cure [3]. It might be expected that HRQoL would worsen over successive LOTs, whereas our findings suggested that HRQoL, functioning, and MM symptoms remained largely consistent across patients in different LOTs. In support of our findings, patients with MM have been reported to experience impaired QoL and elevated psychological distress independent of LOT (1L–4L +) [24]. One possible factor contributing to the lack of progressively worse HRQoL with increasing LOTs is the expanding availability of novel therapies for use in later lines, where treatment options in later stages of treatment may previously have been limited [4, 6]. However, it is noteworthy that we found clinically meaningful differences for global health status and physical functioning with worse scores in patients in 4L than in 1L.

Aligning treatment preferences between patients with MM and their physicians may be challenging, particularly in patients who are relapsed or refractory to multiple therapies [25, 26]. A key driver of discordance between patients and physicians could be differences in priorities for treatment outcomes. Previous studies show that increasing survival is the most important outcome of treatment for both patients and physicians [11], but patients also place high importance on other factors, such as avoiding treatment side effects [12]. In the present study, HRQoL appeared worse in patients who reported higher levels of side-effect bother, suggesting that perception of treatment side effects influenced HRQoL. This finding is consistent with a pooled analysis of 5765 patients with a range of primary cancers across 4 real-world studies and clinical trials, which also found that EQ-5D-3L scores decreased with increasing FACT-GP5 ratings [27]. Furthermore, a previous study examining HRQoL in elderly patients with any type of cancer reported that fatigue, social functioning, and burden of illness had a strong association with global HRQoL [28]. Indeed, a systematic review of factors influencing older patients’ decision to accept or decline treatment identified fear of side effects as a common reason for declining treatment [29]. The influence of patient perception of treatment side effects on HRQoL, beyond the inherent impact of side effects, indicates the importance of patient perception in the assessment of treatment burden and patient-physician communication in optimizing treatment outcomes [30].

In contrast, poor patient-physician communication can lead to delayed or suboptimal detection of side-effect incidence and severity, with potential consequences for treatment adherence, symptom control, HRQoL, and survival [31, 32]. We found a poor to fair agreement in the frequency of reported side effects between patients and physicians, with physicians reporting some categories of side effects at lower rates than patients. This finding is supported by research that investigated toxicity reporting in three clinical trials, in which agreement between patients with breast or non–small-cell lung cancer and physicians was low for all toxicities. Comparison of investigator-completed case report forms with patient-reported EORTC QoL questionnaires demonstrated investigator under-reporting of toxicities by 41–74%, even within the clinical trial setting [33]. Similarly, a comparison of palliative care patient-reported EORTC QLQ-C30 with a proxy QoL questionnaire completed by physicians has illustrated that physicians’ perception of their patients’ HRQoL may be clinically different from the patients’ own assessment [34]. A study evaluating self-reported pain also showed a discordance between the perceptions of MM patients compared with physicians, with nearly half of physicians underestimating bone pain severity, possibly reflecting the lack of time, experience, and tools available to physicians to assess the full impact of symptoms on patients [35]. Furthermore, differences in patient and physician perspectives may also be influenced by patients’ level of health literacy, which can in turn be enhanced by improved patient-physician communication [6, 32, 36]. Together, this highlights a need for effective patient-physician communication, and potentially the inclusion of PRO tools into treatment decisions to ensure accurate reporting and management of treatment side effects, and to optimize HRQoL outcomes for patients.

Certain limitations should be considered when interpreting our results. The survey reflected the study population at a single time point rather than longitudinally; while data stratified by LOT were reported, they did not represent changes in HRQoL over time. Use of treatment regimens was evaluated rather than individual drugs, and treatment side effects were grouped for analysis; this meant that side effects could not be attributed to the specific treatment regimen each patient was receiving at the time of data collection. Additionally, certain types of symptoms and treatment side effects may have been difficult for patients to evaluate and report. For example, hematological side effects may have been under-reported by patients due to their lower ability to perceive the impact compared with other types of side effects. Together with level of health literacy, this further supports the importance of good patient-physician communication to help patients understand the impact of medications they are prescribed, including the side effects listed in the patient information sheets that accompany their prescribed medications. The patient sample reflected actively treated patients and may not have been representative of the broader MM population. There were no patients within the patient record form sample who were on best supportive care only, receiving a “watch and wait” treatment approach, or currently enrolled in clinical trials. The data collected in the survey were based on a convenience sample, and the quality of data depended on the accurate reporting of information by physicians and patients. However, the patient sample was representative of how patients with MM are treated in the real world.

This single time point survey was strengthened by the analysis of data collected over several time points from patients’ medical records, including at diagnosis, at each LOT, and at the time of the survey. Use of the FACT-GP5 PRO tool in combination with the longstanding and complementary HRQoL metrics provided a detailed analysis of how patients’ perception of side effects influenced HRQoL. Using these tools, we generated valuable insights on various aspects of HRQoL in patients with MM and the extent of concordance between patient and physician reporting of side effects associated with MM treatment in the real world.

## Conclusion

We found that HRQoL of patients with MM was generally consistent between 1 and 4L, although patients in 4L had the worst global health status. HRQoL was also worse with higher levels of patient-reported bother associated with treatment side effects. The observed discordance between patient and physician reporting of side effects suggested that certain treatment side effects may have been under-reported by physicians, and those side effects that patients considered bothersome may have been underestimated by their physicians. These findings highlight the importance of effective patient-physician communication to support better identification and management of treatment side effects and, as a result, improvement of HRQoL in patients receiving treatment for MM.

## Supplementary information

Below is the link to the electronic supplementary material.

Supplementary file1 (DOCX 504 KB)

## Data Availability

All data, i.e. methodology, materials, data and data analysis, that support the findings of this survey are the intellectual property of Adelphi Real World. All requests for access should be addressed directly to Amanda Ribbands at Amanda.ribbands@adelphigroup.com. AR is an employee of Adelphi Real World.
