# Supplementary material for: Real-world patient-reported outcomes and concordance between patient and physician reporting of side effects across lines of therapy in multiple myeloma within the USA
Source: Support Care Cancer. 2023 Jun 2;31(6):371. doi: 10.1007/s00520-023-07836-x (PMC10238291; doi:10.1007/s00520-023-07836-x)
Supplement: Supplementary file 1 — Supplementary file1 (DOCX 504 KB) [file 520_2023_7836_MOESM1_ESM.docx]

# Online Resource

**Supplementary Methods**

## Outcomes

Physicians reported patients’ characteristics including demographics, clinical data, treatment history, outcomes associated with each treatment used, and side effects. Patients reported their perceptions of multiple myeloma (MM) treatment and side effects they had experienced, including information on health-related quality of life (HRQoL), global health status, functional scores, body image, MM symptoms, level of side effect bother, perspective on the future, and financial impact of MM. Differences in HRQoL scores between patients at different lines of therapy (LOTs) were assessed with respect to published minimal important differences (MID) for each patient-reported outcome (PRO) measure [1-3]. Changes above MID were considered clinically important.

To assess key aspects of patients’ HRQoL that may have been impacted by MM, patients completed the European Organisation for the Research and Treatment of Cancer Quality of Life Core Questionnaire (EORTC QLQ-C30), a validated 30-item questionnaire designed to assess the HRQoL of patients across 5 functional scales (physical, role, cognitive, emotional, and social) and 3 symptom scales (fatigue, pain, and nausea and vomiting). Single items also assessed global health status, perceived financial impact of the disease, and commonly reported symptoms such as dyspnea, loss of appetite, insomnia, constipation, and diarrhea [4]. Scores ranged from 0–100; higher scores for global health status and functional scales represent better HRQoL and functioning, while higher scores for symptoms scales indicate more severe symptoms. The MID was established to be 5 points [1].

Patients also completed the EORTC MM Module (EORTC QLQ-MY20) to further assess HRQoL. EORTC QLQ-MY20 includes 3 multi-item scales to evaluate disease symptoms, side effects of treatment, and future perspective, and a single item to assess body image [5, 6]. Similar to EORTC QLQ-C30, scores ranged from 0–100 where higher scores for symptoms scales represented more severe symptoms and higher functional scale scores represented better functioning. The MID was established to be 10 points for disease symptoms and side effects, 13 points for body image, and 9 points for future perspectives [2].

To evaluate core dimensions of overall HRQoL and health state, patients completed the descriptive self-assessed EQ-5D-3L [3]. The 5 dimensions assessed were mobility, self-care, usual activities, pain/discomfort, and anxiety/depression. Each dimension was rated on a 3-point scale, indicating no problems, some problems, or extreme problems. Dimension ratings were used to calculate the EQ-5D-3L utility score, which ranged from 0.0 (dead) to 1.0 (full health); a change in score of 0.08 points or more was regarded as clinically meaningful. In addition to the EQ-5D-3L descriptive component, patients rated their overall health state using the Visual Analogue Scale (EQ-5D VAS) with scores ranging from 0 (worst imaginable health state) to 100 (best imaginable health state); the MID was established to be 7 points.

Patients reported the level of bother associated with side effects they experienced as a result of MM treatment using the Functional Assessment of Cancer Therapy - General Population (FACT-GP) questionnaire. FACT-GP is a 21-item assessment of four basic domains of self-assessed well-being: physical, social/family, emotional, and functional [7]. In this study, item 5 on the physical well-being scale (FACT-GP5) was used to assess side-effect bother on a 5-point Likert scale from 0 (“not at all” bothered) to 4 (“very much” bothered) [8]. FACT-GP5, due to its significant association with clinician-reported adverse events and patients’ general ability to enjoy their lives, provides an overall summary measure of the burden of a given set of treatments [9].

# References

1. Kvam AK, Fayers P, Wisloff F (2010) What changes in health-related quality of life matter to multiple myeloma patients? A prospective study. Eur J Haematol 84:345–53

2. Sully K, Trigg A, Bonner N, et al (2019) Estimation of minimally important differences and responder definitions for EORTC QLQ-MY20 scores in multiple myeloma patients. Eur J Haematol 103:500–9

3. Pickard AS, Neary MP, Cella D (2007) Estimation of minimally important differences in EQ-5D utility and VAS scores in cancer. Health Qual Life Outcomes 5:70

4. Velikova G, Coens C, Efficace F, et al (2012) Health-Related Quality of Life in EORTC clinical trials — 30 years of progress from methodological developments to making a real impact on oncology practice. EJC Suppl 10:141

5. Reilly MC, Zbrozek AS, Dukes EM (1993) The validity and reproducibility of a work productivity and activity impairment instrument. Pharmacoeconomics 4:353–65

6. Stead ML, Brown JM, Velikova G, et al (1999) Development of an EORTC questionnaire module to be used in health-related quality-of-life assessment for patients with multiple myeloma. European Organization for Research and Treatment of Cancer Study Group on Quality of Life. Br J Haematol 104:605–11

7. Lindqvist Bagge AS, Carlander A, Fahlke C, et al (2020) Health-related quality of life (FACT-GP) in Sweden. Health Qual Life Outcomes 18:172

8. Griffiths P, Peipert JD, Leith A, et al (2022) Validity of a single-item indicator of treatment side effect bother in a diverse sample of cancer patients. Support Care Cancer 30:3613–23

9. Pearman TP, Beaumont JL, Mroczek D, et al (2018) Validity and usefulness of a single-item measure of patient-reported bother from side effects of cancer therapy. Cancer 124:991–7

## Table S1. Patient demographics and clinical characteristics

|  | **N** | **All patients** | | **N** | **Matched patient self-completion questionnaire patients** | |
| --- | --- | --- | --- | --- | --- | --- |
| **Age, years, median (IQR)** | 377 | 69.0 (63.0–74.0) | | 132 | 70.0 (63.2–73.0) | |
| **Sex, n (%)** | 377 |  | | 132 |  | |
| Male |  | 254 (67) | |  | 86 (65) | |
| Female |  | 123 (33) | |  | 46 (35) | |
| **Ethnicity, n (%)** | 377 |  | | 132 |  | |
| White |  | 241 (64) | |  | 93 (70) | |
| African American |  | 94 (25) | |  | 32 (24) | |
| Asian |  | 17 (5) | |  | 1 (1) | |
| Hispanic/Latino |  | 17 (5) | |  | 4 (3) | |
| Other |  | 8 (2) | |  | 2 (2) | |
| **Months since diagnosis, median (IQR)** | 314 | 20.7 (6.5–41.2) | | 117 | 24.3 (5.8–38.4) | |
| **High cytogenic risk, n (%)** | 195 |  | | 67 |  | |
| Yes |  | 76 (39) | |  | 25 (37) | |
| No |  | 119 (61) | |  | 42 (63) | |
| **ECOG-PS at diagnosis,**  **n (%)** | 377 | Diagnosis | Data extraction | 132 | Diagnosis | Data extraction |
| 0 |  | 125 (33) | 76 (20) |  | 43 (33) | 25 (19) |
| 1 |  | 209 (55) | 230 (61) |  | 76 (58) | 82 (62) |
| 2 |  | 30 (8) | 49 (13) |  | 5 (4) | 14 (11) |
| 3 |  | 11 (3) | 18 (5) |  | 8 (6) | 11 (8) |
| 4 |  | 0 (0) | 3 (1) |  | 0 (0) | 0 (0) |
| Unknown |  | 2 (1) | 1 (<1) |  | 0 (0) | 0 (0) |
| **ISS stage at diagnosis,**  **n (%)** | 377 |  | | 132 |  | |
| Monoclonal gammopathy of undetermined significance |  | 4 (1) | |  | 0 (0) | |
| Smoldering multiple myeloma |  | 1 (<1) | |  | 0 (0) | |
| Stage I |  | 77 (20) | |  | 27 (20) | |
| Stage II |  | 144 (38) | |  | 65 (49) | |
| Stage III |  | 130 (34) | |  | 36 (27) | |
| Unknown/not assessed |  | 21 (6) | |  | 4 (3) | |

ECOG-PS, Eastern Cooperative Oncology Group-Performance Status; IQR, interquartile range; ISS, International Staging System.

Table S2. Patient- and physician-reported side effects in total and stratified by LOT

|  | **Total** | | **LOT** | | | | | | | |
| --- | --- | --- | --- | --- | --- | --- | --- | --- | --- | --- |
|  |  |  | **1L** | | **2L** | | **3L** | | **4L** | |
|  | **Physician N=73** | **Patient N=132** | **Physician**  **n=25** | **Patient**  **n=40** | **Physician**  **n=15** | **Patient**  **n=32** | **Physician**  **n=14** | **Patient**  **n=31** | **Physician**  **n=19** | **Patient**  **n=29** |
| **Gastrointestinal** | 40 (55) | 47 (36) | 11 (44) | 15 (38) | 8 (53) | 10 (31) | 6 (43) | 8 (26) | 15 (79) | 14 (48) |
| Nausea | 24 (33) | 33 (25) | 8 (32) | 11 (28) | 5 (33) | 6 (19) | 2 (14) | 6 (19) | 9 (47) | 10 (34) |
| Diarrhea | 16 (22) | 7 (5) | 4 (16) | 1 (2) | 3 (20) | 2 (6) | 1 (7) | 2 (6) | 8 (42) | 2 (7) |
| Constipation | 10 (14) | 19 (14) | 1 (4) | 6 (15) | 1 (7) | 3 (9) | 3 (21) | 3 (10) | 5 (26) | 7 (24) |
| Vomiting | 7 (10) | 11 (8) | 0 (0) | 2 (5) | 1 (7) | 4 (12) | 0 (0) | 1 (3) | 6 (32) | 4 (14) |
| **Blood/circulatory** | 27 (37) | 14 (11) | 3 (12) | 3 (8) | 7 (47) | 4 (12) | 6 (43) | 1 (3) | 11 (58) | 6 (21) |
| Anemia | 21 (29) | 9 (7) | 2 (8) | 2 (5) | 6 (40) | 3 (9) | 4 (29) | 1 (3) | 9 (47) | 3 (10) |
| Thrombocytopenia | 13 (18) | 2 (2) | 0 (0) | 0 (0) | 3 (20) | 0 (0) | 4 (29) | 0 (0) | 6 (32) | 2 (7) |
| Neutropenia | 5 (7) | 3 (2) | 1 (4) | 1 (2) | 0 (0) | 0 (0) | 2 (14) | 0 (0) | 2 (11) | 2 (7) |
| **Neuro/psychological conditions** | 24 (33) | 36 (27) | 5 (20) | 8 (20) | 8 (53) | 9 (28) | 4 (29) | 11 (35) | 7 (37) | 8 (28) |
| Neuropathy | 20 (27) | 18 (14) | 5 (20) | 4 (10) | 7 (47) | 4 (12) | 3 (21) | 6 (19) | 5 (26) | 4 (14) |
| Depression | 4 (5) | 18 (14) | 0 (0) | 4 (10) | 0 (0) | 7 (22) | 1 (7) | 3 (10) | 3 (16) | 4 (14) |
| Mood changes | 1 (1) | 14 (11) | 0 (0) | 4 (10) | 1 (7) | 0 (0) | 0 (0) | 6 (19) | 0 (0) | 4 (14) |
| **Dermatological** | 11 (15) | 22 (17) | 1 (4) | 3 (8) | 2 (13) | 6 (19) | 2 (14) | 7 (23) | 6 (32) | 6 (21) |
| Dry skin | 7 (10) | 19 (14) | 1 (4) | 3 (8) | 0 (0) | 5 (16) | 1 (7) | 6 (19) | 5 (26) | 5 (17) |
| Rash | 8 (11) | 6 (5) | 1 (4) | 0 (0) | 2 (13) | 1 (3) | 1 (7) | 2 (6) | 4 (21) | 3 (10) |
| **Other** | 37 (51) | 80 (61) | 13 (52) | 29 (72) | 7 (47) | 16 (50) | 8 (57) | 17 (55) | 9 (47) | 18 (62) |
| Fatigue/tiredness | 26 (36) | 57 (43) | 10 (40) | 25 (62) | 5 (33) | 10 (31) | 6 (43) | 10 (32) | 5 (26) | 12 (41) |
| General aches and pains | 1 (1) | 22 (17) | 0 (0) | 4 (10) | 0 (0) | 3 (9) | 0 (0) | 5 (16) | 1 (5) | 10 (34) |
| Headaches | 2 (3) | 15 (11) | 1 (4) | 6 (15) | 0 (0) | 3 (9) | 0 (0) | 2 (6) | 1 (5) | 4 (14) |
| Blurred vision | 3 (4) | 9 (7) | 0 (0) | 2 (5) | 1 (7) | 2 (6) | 0 (0) | 2 (6) | 2 (11) | 3 (10) |
| Fever/flu-like symptoms | 1 (1) | 7 (5) | 1 (4) | 3 (8) | 0 (0) | 3 (9) | 0 (0) | 0 (0) | 0 (0) | 1 (3) |
| Loss of appetite | 7 (10) | 0 (0) | 2 (8) | 0 (0) | 0 (0) | 0 (0) | 2 (14) | 0 (0) | 3 (16) | 0 (0) |
| Weight loss | 5 (7) | 0 (0) | 1 (4) | 0 (0) | 1 (7) | 0 (0) | 2 (14) | 0 (0) | 1 (5) | 0 (0) |

Symptoms reported by ≥5% of patients or physicians presented. All data are reported as n (%).

1L, first LOT; 2L, second LOT; 3L, third LOT; 4L, fourth LOT; LOT, line(s) of therapy.

Table S3. Patient-reported side effects stratified by FACT-GP5 bother rating

|  | Bothersome side effects (from the FACT-GP5) | | | |
| --- | --- | --- | --- | --- |
| **Side effect, n (%)** | Total | 0  Not at all | 1–2  A little bit/somewhat | 3–4  Quite a bit/very much |
| **Fatigue/tiredness** | 57 (100) | 2 (4) | 44 (77) | 11 (19) |
| **Nausea** | 33 (100) | 1 (3) | 21 (64) | 11 (33) |
| **General aches and pains** | 22 (100) | 3 (14) | 11 (50) | 8 (36) |
| **Constipation** | 19 (100) | 0 (0) | 9 (47) | 10 (53) |
| **Dry skin** | 19 (100) | 1 (5) | 11 (58) | 7 (37) |
| **Neuropathy** | 18 (100) | 1 (6) | 12 (67) | 5 (28) |
| **Depression** | 18 (100) | 0 (0) | 8 (44) | 10 (56) |
| **Headaches** | 15 (100) | 1 (7) | 8 (53) | 6 (40) |
| **Mood changes** | 14 (100) | 1 (7) | 5 (36) | 8 (57) |
| **Vomiting** | 11 (100) | 0 (0) | 7 (64) | 4 (36) |
| **Anemia** | 9 (100) | 0 (0) | 6 (67) | 3 (33) |
| **Blurred vision** | 9 (100) | 0 (0) | 3 (33) | 6 (67) |
| **Diarrhea** | 7 (100) | 0 (0) | 2 (29) | 5 (71) |
| **Fever/flu-like symptoms** | 7 (100) | 0 (0) | 3 (43) | 4 (57) |
| **Rash** | 6 (100) | 2 (33) | 0 (0) | 4 (67) |
| **Flushing** | 5 (100) | 0 (0) | 0 (0) | 5 (100) |
| **Sensitivity to light** | 5 (100) | 1 (20) | 1 (20) | 3 (60) |
| **Neutropenia** | 3 (100) | 0 (0) | 2 (67) | 1 (33) |
| **Kidney insufficiency** | 3 (100) | 0 (0) | 1 (33) | 2 (67) |
| **Dry eye** | 3 (100) | 0 (0) | 2 (67) | 1 (33) |
| **Thrombocytopenia** | 2 (100) | 0 (0) | 1 (50) | 1 (50) |
| **Thrombosis** | 2 (100) | 0 (0) | 0 (0) | 2 (100) |
| **Cardiovascular event** | 1 (100) | 0 (0) | 1 (100) | 0 (0) |
| **Leucopenia** | 1 (100) | 0 (0) | 0 (0) | 1 (100) |
| **None of the above** | 24 (100) | 15 (62) | 8 (33) | 1 (4) |

Data presented from all patients who completed the FACT-GP5 in patient self-completion questionnaire.

FACT-GP5, Functional Assessment of Cancer Therapy - General Population item 5.

Figure S1. Linear regression analysis of EORTC QLQ-C30 functional scales


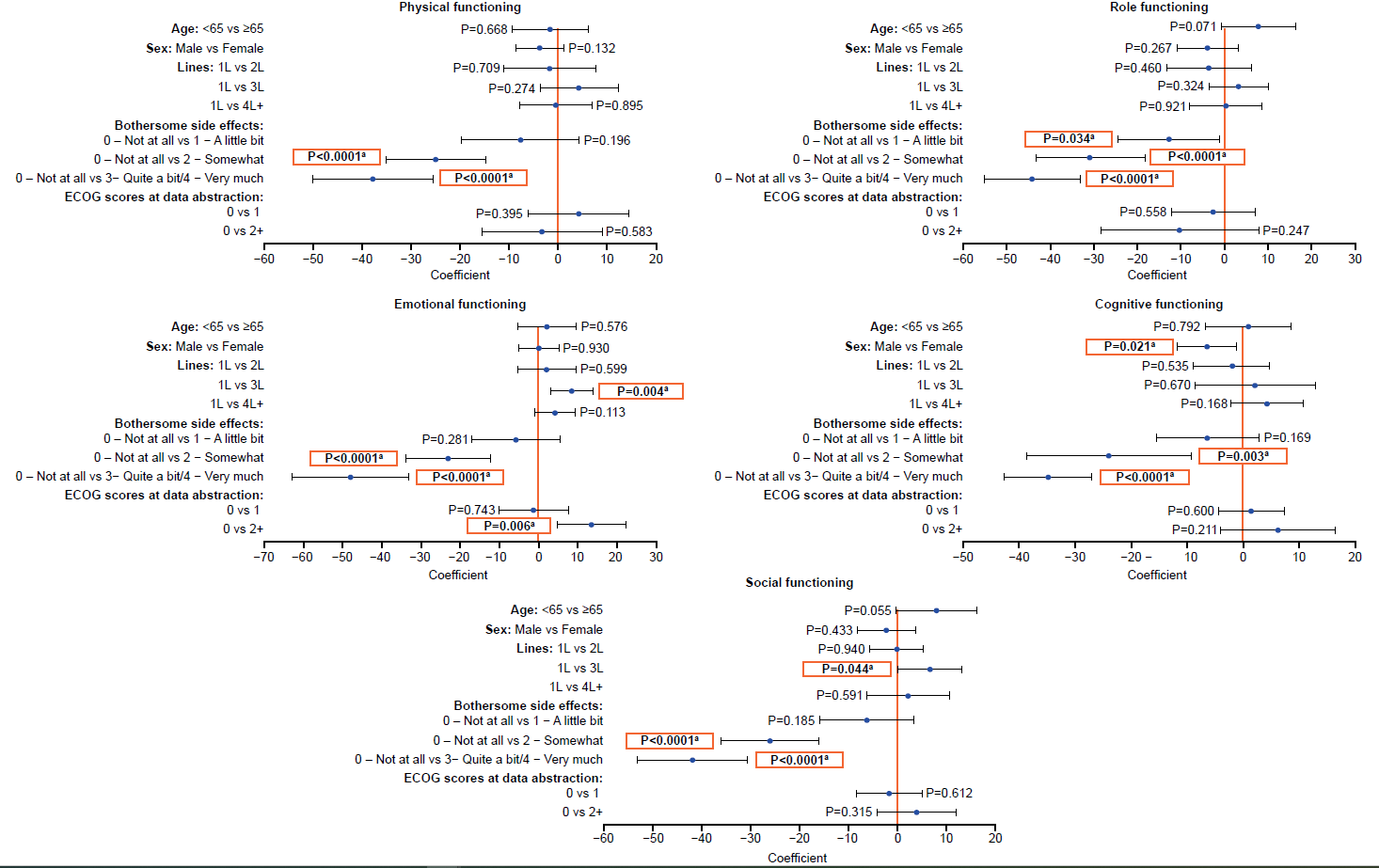


Data are presented as the estimate coefficient ±95% CI for each covariate. ^a^Indicates statistically significant P-value. Significant values <0 indicate a higher likelihood of having a lower score for the relevant scale.

1L, first LOT; 2L, second LOT; 3L, third LOT; 4L, fourth LOT; CI, confidence interval; ECOG, Eastern Cooperative Oncology Group; EORTC QLQ-C30, European Organisation for the Research and Treatment of Cancer Quality of Life Core Questionnaire; LOT, line(s) of therapy.

Figure S2. Linear regression analysis of EORTC QLQ-MY20 scales


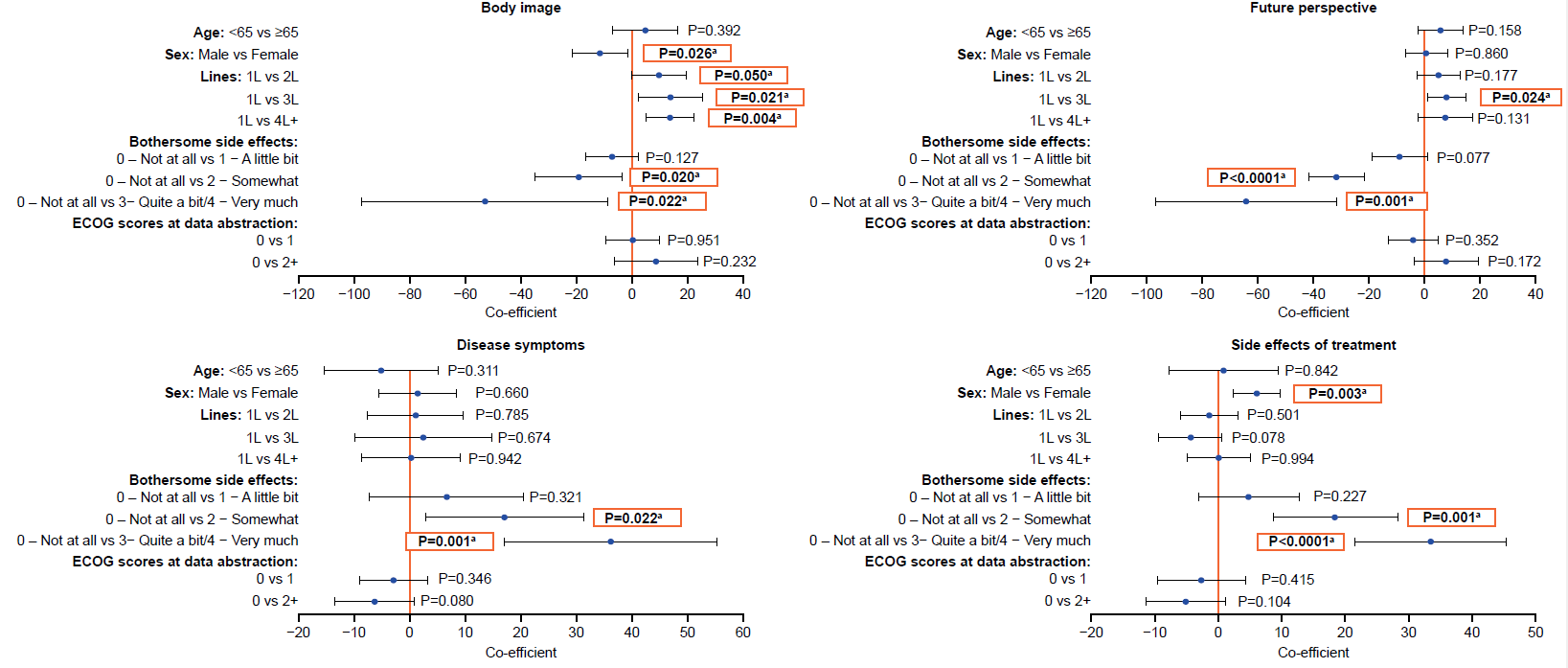


Data are presented as the estimate coefficient ±95% CI for each covariate. ^a^Indicates statistically significant P-value. Significant values <0 indicate a higher likelihood of having a lower score for the relevant scale.

1L, first LOT; 2L, second LOT; 3L, third LOT; 4L, fourth LOT; CI, confidence interval; ECOG, Eastern Cooperative Oncology Group; EORTC QLQ-MY20, European Organisation for the Research and Treatment of Cancer Quality of Life Core Questionnaire multiple myeloma module; LOT, line(s) of therapy.

Figure S3. Patient- vs physician-reported symptoms at time of data collection for each LOT analysis of EORTC QLQ-C30 functional scales


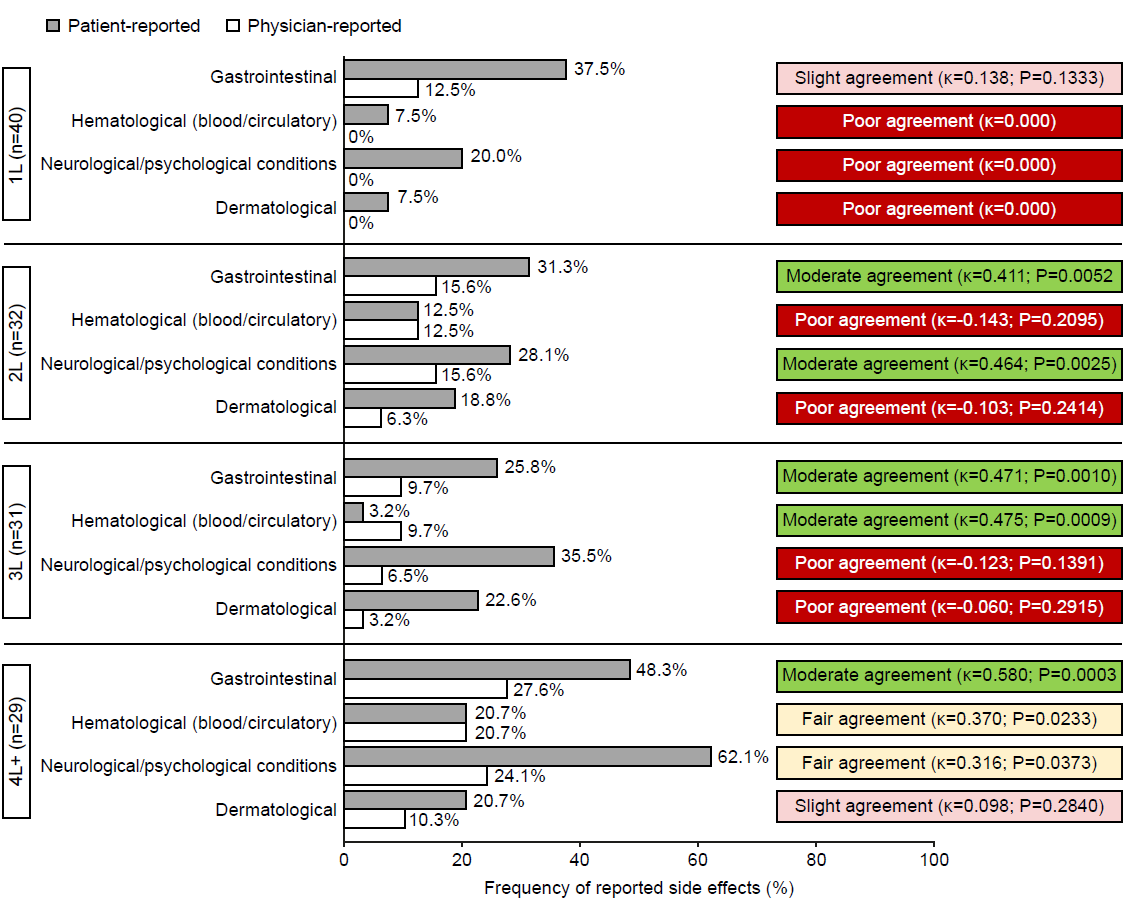


Data show number of patient self-completion questionnaires and physician-reported patient record forms reporting each class of side effects.

Extent of agreement was determined using Cohen’s kappa coefficient (κ).

1L, first LOT; 2L, second LOT; 3L, third LOT; 4L, fourth LOT; EORTC QLQ-C30, European Organisation for the Research and Treatment of Cancer Quality of Life Core Questionnaire; LOT, line(s) of therapy.
